# 1897—Journal—1898

**Published:** 1897-12

**Authors:** 


### EDITORIAL.

### 1897—JOURNAL—1898.

WITH this number we close the eighteenth volume of the JOURNAL OF COMPARATIVE MEDICINE AND VETERINARY ARCHIVES. 1897 has been a year of uncertainty in business circles, and to the veterinary profession, whose usefulness is so largely commercial, it has been one of great doubt and disappointment. The JOURNAL has had a more prosperous year than it had in 1896, for which we recognize our indebtedness for the great help and assistance afforded us by the members of the profession. We have been favored with more valuable material than in any one year of our history, and we have rendered a full account to our readers. In addition to the sixty-four well-filled pages each month, we have added some two hundred and thirteen extra pages—or an average of nearly twenty to each issue during 1897. Every movement for the well-being and advancement of the profession has received our unqualified support. Association circles everywhere have felt the strength and support of the JOURNAL. Control work all over the land has been carefully followed and our readers kept informed of every important movement. We have kept in touch with the work and movements of the profession at home, and noted much of those in foreign lands. We have thus contributed to the common bond of union that draws us together. We have been open and fearless in our editorial opinions, and have had no enemies to punish, no friends to reward, other than where it was for the good of the whole profession.

We hasten the outgoing of the old year, believing that in welcoming the new we are assured of a greater measure of good things for our readers and subscribers than the past year has brought them. The better condition of our agricultural population and the revival of the live-stock industry are sure to be noted during the coming year, and the increased interest in the sanitary fields of the veterinarian is sure to bring an increased measure of prosperity to all. Never in the JOURNAL'S history has it been prepared to enter a new year with as great a corps of assistants and contributors as it will for 1898. The ablest writers in the land, the largest corps of contributors, the most extensive list of correspondents, will keep our columns well filled with information of every kind, to instruct, to enlighten, to interest, and to strengthen fraternal feelings one with another, and thus equip the largest number in the most thorough manner for every aspect of our work. We thank all of those who have so ably aided us the past year, and we wish for all in the new year an era of prosperity that has not been possible for several years. Speed the outgoing, welcome the incoming.

